# Epidemiology of Toxoplasmosis in SERBIA: A Cross-Sectional Study on Blood Donors

**DOI:** 10.3390/microorganisms10030492

**Published:** 2022-02-23

**Authors:** Milena Stopić, Tijana Štajner, Ljiljana Marković-Denić, Vladimir Nikolić, Iva Djilas, Snežana Jovanović Srzentić, Olgica Djurković-Djaković, Branko Bobić

**Affiliations:** 1 National Reference Laboratory for Toxoplasmosis, Group for Microbiology and Parasitology, Center of Excellence for Food- and Vector-Borne Zoonoses, Institute for Medical Research, National Institute of Republic of Serbia, University of Belgrade, 11129 Belgrade, Serbia; milenastopic@yahoo.com (M.S.); olgicadj@imi.bg.ac.rs (O.D.-D.); bobicb@imi.bg.ac.rs (B.B.); 2 Institute of Epidemiology, Faculty of Medicine, University of Belgrade, 11000 Belgrade, Serbia; markovic.denic@gmail.com (L.M.-D.); nikolicvladimir32@gmail.com (V.N.); 3 Blood Transfusion Institute of Serbia, 11000 Belgrade, Serbia; ivaiva@gmail.com (I.D.); srzentics60@yahoo.com (S.J.S.)

**Keywords:** *Toxoplasma gondii*, toxoplasmosis, prevalence, risk factors, blood donors

## Abstract

Toxoplasmosis is a globally distributed parasitic zoonosis, affecting approximately one third of the human population. Epidemiological studies on toxoplasmosis conducted in Serbia so far have been focused on women of childbearing age, without a clear insight into the prevalence in the general population. We conducted a cross-sectional study in a representative sample of the healthy adult population consisting of 1095 blood donors of both genders to establish the prevalence and risk factors for *Toxoplasma gondii* infection. Data on the demographic and clinical characteristics of all study participants, as well as on their lifestyle habits, were collected by means of a questionnaire. The overall prevalence of infection was 20.5% (224/1095) and the avidity of the specific IgG antibodies detected was high in a vast majority of the seropositive donors (98.2%). Interestingly, the remaining 1.8% of the specific IgG positive samples were of borderline avidity (4/224), in complete absence of specific IgM. The multivariate logistic regression analysis showed that independent risk factors included age (from OR (95% CI) 1.9 (1.13–3.28) in the 30–39 age group, to 6.8 (3.27–14.24) in the age group of >60 years), suburban living (OR (95% CI) 2.2 (1.43–3.34)) and contact with soil (OR (95% CI) 1.4 (1.01–1.94)). This first large-scale study on toxoplasmosis in the general population in Serbia shows the lowest prevalence ever reported in this country. Moreover, the novel perspective on risk factors provides an updated basis for future prevention programs.

## 1. Introduction

Toxoplasmosis is a worldwide omnipresent parasitic disease, caused by the obligately intracellular protozoan *Toxoplasma gondii.* The life cycle of *T. gondii* involves members of the Felidae family, as the definitive host, and warm-blooded animals, including humans, as intermediate hosts. Sexual reproduction occurs only in the intestines of the Felidae, ending in excretion of oocysts with the feces into the environment. Humans become infected mainly by consumption of water or fruits and vegetables contaminated with oocysts, or by ingestion of tissue cysts in the undercooked or raw meat of infected animals [1]. Even though viable parasites may be transmitted via the placenta or transplanted organs, the possibility of transmission via blood transfusion is insufficiently documented, hence neglected. Reports on transmission of *T. gondii* by leukocyte or platelet transfusion are rare, though the parasite can preserve its viability for up to 50 days in citrated blood at 5 °C [2,3,4].

Toxoplasmosis in immunocompetent individuals is generally asymptomatic or presents with flu-like symptoms. In contrast, it is most detrimental for the fetus and immunosuppressed patients [5]. Clinical manifestations in immunocompromised individuals are generally severe and possibly life-threatening. This is particularly important in view of the global surge in transplantation medicine, which leads to an increasing number of immunosuppressed patients of both genders potentially endangered by *T. gondii*, graft-acquired or reactivated [6]. 

It has been estimated that one-third of the human population is chronically infected with this parasite [7]. The prevalence of toxoplasmosis varies not only among countries but also among regions within one country and these variations are a product of climatic, geographic and cultural differences. However, over the past decades, the number of seropositive individuals has been decreasing globally. In line with this global trend, the prevalence of toxoplasmosis in southern and southeastern European countries, including Serbia, has been continuously declining [8]. However, most data for Serbia have resulted from studies conducted in women of childbearing age [9] and did not afford insight into the status of *T. gondii* infection in the general population.

Therefore, we conducted a comprehensive epidemiological study on a representative sample of the adult general population, as a prerequisite for planning locally efficient future prevention programs for toxoplasmosis.

## 2. Materials and Methods

### 2.1. Study Population and Data Collection

This epidemiological cross-sectional study included a total of 1095 healthy participants presenting for blood donation at the Blood Transfusion Institute of Serbia between December 2017 and July 2018. The participants included donors of both genders, aged from 18 to 65, residing in the Belgrade area. The participants were recruited according to a pre-formed plan to include both genders and all age groups as evenly as possible; however, due to a low rate of blood donation by individuals above the age of 60, these ended up under-represented in the final sample. 

Physical examination of the donors; blood sampling; routine laboratory screening for HIV, hepatitis B, hepatitis C and syphilis; and donor interview were conducted at the Blood Transfusion Institute of Serbia, while the analytical part of the study (research planning, diagnosis of toxoplasmosis and data analysis) was performed at the National Reference Laboratory for Toxoplasmosis, Institute for Medical Research, University of Belgrade.

The donors were interviewed by physicians using an epidemiological questionnaire specifically designed for this study. The questionnaire was divided into sections; (1) basic demographic data collected included gender, age (stratified in 5 groups, 18–29, 30–39, 40–49, 50–59 and >60 years), occupation, level of education (elementary school, high school and university), residence area (urban or suburban areas of Belgrade) and type of housing (townhouse with garden or flat in building); (2) lifestyle data collected included consumption of raw or undercooked meat, contact with soil (gardening or farming) and contact with cats (cat ownership and/or contact with stray cats); (3) medical history data collected focused on lymphadenopathy (cervical, axillary, or inguinal) and episodes of low-grade fever during the previous 12 months.

All questions, apart from those related to demographic data, were designed to provide answers in dichotomous form (yes or no). 

The study was approved by the Ethical Committees of the Institute for Medical Research (decision number EO122/2017)) and the Blood Transfusion Institute of Serbia (decision number 6270.1). Signed informed consent was a prerequisite for every donor to participate in the study. 

### 2.2. Determining the Status of T. gondii Infection

Serological screening for the presence of specific IgG antibodies was performed by an in-house high sensitivity direct agglutination assay (HSDA) using formalin-fixed tachyzoites of the *T. gondii* RH strain as antigen, as previously described [10]. All sera were serially diluted two-fold starting from 1:20. Visible agglutination at a serum dilution of 1:40 was considered a positive result. According to Desmonts and Remington [10], when compared to the Sabin–Feldman test, the sensitivity of the HSDA was 99.85%, with 98.55% specificity.

Each sample in which specific IgG antibodies were detected by the HSDA was further analyzed by a commercial enzyme immunoassay (VIDAS^®^ Toxo IgG II; TXG, Biomerieux, Lyon, France). The concentration of specific IgG was interpreted according to the manufacturer’s instructions (<4 IU/mL, negative; 4–8 IU/mL, borderline; and ≥8 IU/mL, positive).

All samples with specific IgG ≥ 8 IU/mL were further examined for their avidity, using the VIDAS^®^ Toxo IgG avidity assay (TXGA, Biomerieux, Lyon, France). The result was expressed as an avidity index (IA) and interpreted according to the manufacturer’s instructions, noting that high IA excluded *T. gondii* infection in the last 4 months (IA < 0.2, low avidity; IA 0.2–0.3, borderline; and IA > 0.3, high avidity).

In case TXGA showed low or borderline avidity, specific IgM antibodies were determined by an enzyme immunoassay (VIDAS^®^ Toxo IgM; TXM, Biomerieux, Lyon, France), in order to confirm or exclude acute infection. The result was expressed in the form of an index (I) and interpreted according to the manufacturer’s instructions (I < 0.55, negative; 0.55–0.65, borderline; and >0.65, positive).

The manufacturer (Biomerieux, Lyon, France) indicated the relative performance data (comparing the assay results with those of a similar assay or a composite reference method) for VIDAS^®^ Toxo IgG II (sensitivity, 98.35%; specificity, 99.77%), VIDAS^®^ Toxo IgG avidity assay (sensitivity, 96.5%; specificity, 87.6%) and VIDAS^®^ Toxo IgM (sensitivity, 97.7%; specificity, 99.4%).

### 2.3. Statistical Data Analysis

Descriptive and analytical statistical methods were used in data processing. Data are presented as mean ± SD or median and number (percentage) for categorical variables. A chi-squared or Fisher’s exact test was used for the analysis of categorical data, as appropriate. *T. gondii* infection risk factors were analyzed by logistic regression. Variables that were significantly associated with *T. gondii* infection in univariate logistic regression analyses at a significance level of *p* < 0.1 were entered into the multivariable logistic regression model. Odds ratios (OR) with 95% confidence intervals (CI) were computed and the Hosmer–Lemeshow goodness-of-fit test was performed to assess overall model fit. The statistical analyses were performed using SPSS version 23.0 software (SPSS Inc., Chicago, IL, USA).

## 3. Results

Specific IgG antibodies were detected in 224/1095 healthy blood donors, indicating an overall prevalence of *T. gondii* infection of 20.5%. The gender distribution among the donors was almost equal, as women accounted for 49.4% (541) and men for 50.6% (554). All blood samples positive for *T. gondii* antibodies in HSDA were also positive in the Toxo IgG II assay and these antibodies were of high avidity in 98.2% (220/224) of the donors. In all four remaining donors (1.8%), in which IgG avidity was not high, it was borderline (not low) and specific IgM antibodies were not detected in any of these samples. The mean concentration of specific IgG detected in this study was 82.01 ± 97.597.

**Figure 1:**
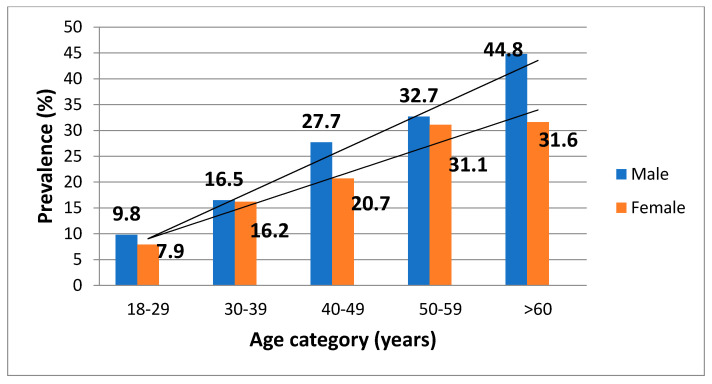
Prevalence of *T. gondii* infection in female and male donors according to age category.

Basic demographic data alongside univariate logistic regression results for *T. gondii* infection are presented in Table 1. Based on these data, older age was associated with a greater chance of being infected by *T. gondii*. Furthermore, the majority of the infected donors was in the older age categories; there was a gradual and continuous increase in the prevalence, ranging from 8.7% in the youngest to 39.6% in the oldest age categories (Table 1). Gender was not associated with *T. gondii* infection (Table 1). However, a detailed analysis revealed a linear increase in the prevalence of infection with age in the population of female donors (*p* < 0.001) (Figure 1). Thus, women of childbearing age were at greater risk of acquiring toxoplasmosis than older women, who were more likely to be already infected (*p* < 0.001). Similarly, male donors in the older age groups were more likely to be infected than the younger ones (Figure 1). In addition, male donors living in the suburban communities of the city were more likely to be infected than those living in the urban ones (*p* < 0.001), as were men working with soil (usually farmers) (*p* < 0.001).

In fact, all donors (irrespective of gender) living in the peripheral communities of Belgrade were more likely to be infected than those living in the central, urban areas of the city (OR 2.1; 95% CI, 1.31–3.41) (Table 1).

Table 2 presents data related to the lifestyle habits that could be associated with contracting *T. gondii* infection. Interestingly, contact with soil (gardening or farming) was significantly associated with infection (OR 1.9; 95% CI, 1.38–2.56). On the other hand, neither contact with cats nor consumption of raw/undercooked meat were associated with *T. gondii* infection in the examined population.

Medical history data, such as lymphadenopathy and low-grade fever during the previous year, were not associated with *T. gondii* infection (*p* = 0.282 and *p* = 0.099, respectively).

Further analyses based on a multivariate regression analysis confirmed all factors associated with infection in the univariate analysis and entered in the final multivariate model, including older age, residing in a suburban area and contact with soil as highly significant predictors of *T. gondii* infection (Table 3).

## 4. Discussion

This is the first epidemiological study on toxoplasmosis in the population of blood donors in Serbia and among only a few in Europe, providing a valuable novel insight into the infection prevalence in both genders and showing alterations in the significance of common risk factors for *T. gondii* infection compared to previous studies. 

The decade-long trend of a steady decline in the prevalence of toxoplasmosis in Europe [11,12,13] has also been shown in the countries of southeastern Europe (including the Balkans). For instance, the prevalence of toxoplasmosis in women of childbearing age in Slovenia decreased from 52% to 25% in barely two decades’ time [8]. This decline in the prevalence was even more striking in women in Serbia—from 85% in 1988 to 31% in 2007 [14,15]. Since there is no systematic screening program for toxoplasmosis in Serbia, those data were based on selected groups of women of childbearing age women diagnosed for toxoplasmosis upon clinical indications (pregnancy-related pathology, lymphadenopathy, etc.). The last study based on a sample of the general population was conducted over 50 years ago [16] and an update was urgently needed.

Blood donors are a part of the adult population in good general health with a practically equal distribution of both genders. According to worldwide data, one third of them (33%) may be considered to be chronically infected with *T. gondii* [17], but the prevalence of infection varies greatly among countries. However, the overall prevalence of toxoplasmosis of 20.5% detected in this study is much lower than that reported among blood donors in the neighboring countries—Croatia (47.8% and 53.8%) [18] and Bosnia and Herzegovina (30.6%) [19]—in the past decade. The diagnostic algorithm for toxoplasmosis and methodology applied in our study is widely used in Reference Laboratories for Toxoplasmosis throughout Europe. The prevalence data from neighboring Croatia were based on a sample of 219 voluntary blood donors (nearly five times smaller than our study sample), while the methodology was comparable to ours (ELISA for specific IgG, IgM and IgA antibodies) [18]. Accordingly, the Bosnia and Herzegovina study was based on 320 blood donors (over three times less than in our study) and the diagnostic was performed in our lab, using HSDA (initial test in our study). The possibility of methodological bias in our study is bypassed by performing two methodologically different tests for specific IgG—one based on direct agglutination (HSDA) and one ELISA-based (VIDAS Toxo IgG II). More likely, the differences in prevalence between our and the studies performed in these neighboring countries are due to the differences in the size of the study samples and the fact that they were performed more than 12 and 6 years ago, respectively [18,19]. Nowadays, the results in these countries would probably also present a declining prevalence.

In fact, to our knowledge, this is the lowest prevalence of *T. gondii* infection in blood donors in Europe, while lower rates were reported in South American (Mexico, 13.5%) and Asian (China, 4.8%) countries [20,21]. We believe that it could be the result of increased public awareness due to health education (especially wide availability of internet-based data on toxoplasmosis), improved hygiene conditions on animal farms and extensive use of frozen meat and vegetables (with freezers in almost every household). In fact, the majority of the population, especially in major cities such as Belgrade is focused on supermarkets for grocery shopping, either for industrially processed meat products and frozen meat or pre-washed vegetables and fruits. It is why we believe that the data represented in this study should be supplemented by data on prevalence of toxoplasmosis in residents of predominantly rural regions of the country who are still largely oriented towards local farmers’ markets or household production of food and food products for domestic use. Even though undercooked meat consumption is no longer identified as a significant risk factor for toxoplasmosis, the prevalence continues to decrease, such as in Greece and Northern Macedonia, where consumption of undercooked meat, as in our study, was not found to be a risk factor [8].

In accordance with previous studies conducted on blood donors [19,22], we have also found that the prevalence increases with age, obviously as a result of the cumulative effect of exposure to the parasite over the years. This increase in the infection prevalence is linear, but, unlike the Bosnia and Herzegovina study [19], in this study, it was detected in both genders, ranging from 8.7% in the youngest age group (18–29 years) to 39.6% in the oldest (>60 years). For the first time in Serbia, we analyzed the prevalence of *T. gondii* infection in healthy adult males and showed it has matched or surpassed the prevalence in female donors of the same age, albeit not significantly. Interestingly, a similar observation arose in a study conducted in Croatia, with the infection being slightly more prevalent in male than female blood donors (53.8% vs. 47.8%) [18]. The demonstration that the prevalence of infection in both males and females rises with age is significant since the potential for drug-induced immunosuppression (e.g., due to malignant disorders, as indications for HSCT or SOT) also increases as people age. The results in males are important, as the male population has rarely been studied and both genders are equally exposed to potentially life-threatening disease in case of immunosuppression [23].

Furthermore, although the majority of immunosuppressed (and virtually all transplanted) patients require regular blood transfusions during their long-term treatment, neither blood units nor blood components are routinely tested for the presence of *T. gondii* or *T. gondii*-specific antibodies in Serbia [24]. However, *T. gondii* tachyzoites may be transmitted from an acutely infected donor (in the stage of parasitemia) to the recipient, which is all the more concerning knowing that the majority of *T. gondii*-infected immunocompetent adults are asymptomatic even during parasitemia [25]. Nevertheless, not a single case of acute infection was detected among more than a thousand blood donors included in this study, suggesting that the risk of parasite transmission via blood transfusion is low. Of the 224 donors with detectable specific IgG only four (1.8%) were of borderline IgG avidity, but the complete absence of specific IgM ruled out acute infection, hence parasitemia. Although high IgG avidity rules out acquired infection within the last 4 months, a borderline or low avidity result does not necessarily mean that the patient has a recently acquired infection, since these results may persist for as long as 1 year (or even longer). Indeed, results indicating a slow maturation of specific IgG avidity have been observed in a substantial number of patients (15.4%, in our previous study), months or even years after the acute infection [26]. Even though avidity testing may be very helpful when the diagnosis is based on a single sample, it should not be used alone as a definitive test for decision making. Accordingly, the proper diagnosis of toxoplasmosis should be based on a panel of serological tests aimed at the determination of specific IgG, specific IgM and specific IgG avidity at least, routinely performed at the Serbian National Reference Laboratory for Toxoplasmosis. The high avidity of the specific IgG antibodies detected in a vast majority (98.2%) of the donors indicated that the infection was older than 4 months, practically ruling out the presence of circulating parasites at this stage [26]. A minimal risk of *T. gondii* transmission via blood transfusion is also acknowledged by the American Association of Blood Banks (AABB) [27]. 

Among lifestyle risk factors for *T. gondii* infection, this study, for the first time, highlighted contact with soil, either directly via gardening or farming, or by living in a house with a yard, as the leading one in Serbia. However, there were also notions of the significance of soil in our previous studies; epidemiological studies conducted between 1988 and 1997 among women of childbearing age residing in Belgrade indicated contact with soil as a risk factor only in the youngest age category (<20 years) [14,28]. Contact with soil contaminated with *T. gondii* oocysts is a well-known risk factor for *T. gondii* infection [29,30]. Studies have shown, for instance, that pregnant women living in a house with a dirt floor in Mexico [31], children playing in parks in Panama [32] or U.S. farmers [33] were more likely to be infected by *T. gondii* due to frequent and direct contact with the soil. The results of the National Health and Nutrition Examination Survey (NHANES) study conducted in the USA between 1988 and 1994 showed that the prevalence of infection was higher in participants with soil-related jobs than in those in other professions (37% vs. 23.7%) [34]. Contact with soil has also long been recognized as a significant risk factor for toxoplasmosis in Europe, leading to acute *T. gondii* infection in 6–17% of cases in one European multicenter case-control study [35]. 

As in our previous studies based on women of childbearing age [28], this study did not find contact with cats, either domestic or stray, to be a significant risk factor for *T. gondii* infection. Cats are the only possible source of oocysts in the soil [36], but soil contamination with oocysts is determined by the frequency of *T. gondii* infection in cats and the population density of infected animals. Even though no study on the prevalence of infection in cats has been conducted in Serbia so far, our results indicate that they do contaminate the soil to the extent that the soil itself is a significant source of infection. 

The most surprising finding of this study is that consumption of undercooked meat is no longer a significant risk factor for *T. gondii* infection in Serbia, although it has repeatedly been identified as the leading risk factor during the last three decades [14,15,28,37]. This may be attributed to several factors, such as (i) a general improvement of animal husbandry and hygiene conditions on farms, leading to a reduced level of infection in meat animals [38]; (ii) the wide availability of household freezers for long-term storage of fresh meat, which greatly reduces the risk of infection by meat consumption; (iii) increased public awareness of the risk of toxoplasmosis from undercooked meat through various media channels. These have also widely publicized the association of toxoplasmosis with cats, which may have helped adopt safe practices in handling cats, thus diminishing contact with cats as a risk factor for *T. gondii* infection.

Our study has strengths and weaknesses. We have indeed based our study on a representative sample of healthy adults but parts of the general population remained unexamined, namely, the youngest (<18 years) and the oldest citizens (>65 years). Although we did not demonstrate the risk of parasite transmission through blood transfusion, a more extensive study could provide more informative data on this matter. 

On the other hand, this is not only the first study on the prevalence of toxoplasmosis in blood donors conducted in Serbia, but also one of the few in Europe. Based on the results of the presented study obtained in the capital, we can conclude that the prevalence of toxoplasmosis in a healthy adult population is at its lowest since 1969 [16]. This is not surprising in view of the decade-long dramatic decline in the prevalence of toxoplasmosis worldwide, across Europe and in the Balkans. To obtain a better understanding of the infection dynamics and risk factors for human toxoplasmosis, future studies of the general population should include all age categories as well as rural areas of the country.

The presented results may guide future planning of locally oriented prevention programs for toxoplasmosis. Indeed, since the majority of participants in this study had contact with soil through gardening or farming, the importance of using personal protective equipment must be emphasized in every public health education and prevention plan for toxoplasmosis. Information on the benefits of routine use of protective gloves and appropriate hand hygiene after gardening or farming provided by public health officials through media (especially the internet) and conveniently designed brochures, containing selected relevant information on possible impacts of *T. gondii* infection on human health and ways to prevent it. If dissemination is to be carefully considered, preferably through primary health care institutions and professional/trade associations of gardeners/farmers, we believe that the compliance to these preventive measures ought to be noted in future epidemiological studies.

## Figures and Tables

**Table 1:** Demographic characteristics of participants and univariate logistic regression analysis for *T. gondii* infection.

Demographic Characteristics	Total *n* (%)	*T. gondii* Seropositive *n* (%)	Univariate Logistic Regression OR (95% CI)
Age group	1.095 (100)	224 (100)	
18–29 years	263 (24.0)	23 (10.3)	ref.
30–39 years	300 (27.4)	49 (21.9)	1.8 (1.05–3.19)
40–49 years	277 (25.3)	67 (29.9)	3.1 (1.79–5.24)
50–59 years	207 (18.9)	66 (29.5)	4.9 (2.82–8.39)
>60 years	48 (4.4)	19 (8.5)	5.2 (2.15–12.71)
Gender	1.095 (100)	224 (100)	
Female	541 (49.4)	101 (45.1)	
Male	554 (50.6)	123 (54.9)	1.1 (0.76–1.50)
Residence area	1.095 (100)	224 (100)	
Urban	963 (87.9)	180 (80.4)	
Suburban	132 (12.1)	44 (19.6)	2.1 (1.31–3.41)
Level of education	999 (100)	196 (100)	
Elementary school	58 (5.8)	15 (7.7)	ref.
High school	520 (52.1)	114 (58.2)	0.9 (0.50–1.82)
University	421 (42.1)	67 (34.2)	0.8 (0.42–1.66)
Type of housing	1.092 (100)	224 (100)	
Townhouse with garden	422 (38.6)	99 (44.2)	0.9 (0.66–1.31)
Flat in building	670 (61.4)	125 (55.8)	

**Table 2:** Lifestyle habits of participants and univariate logistic regression for *T. gondii* infection.

Lifestyle Habits	Total *n* (%)	*T. gondii* Seropositive *n* (%)	Univariate Logistic Regression OR (95% CI)
Contact with soil	1.090 (100)	222 (100)	
No	777 (71.3)	134 (60.4)	
Yes	313 (28.7)	88 (39.6)	1.9 (1.38–2.56)
Contact with cat	1.095 (100)	224 (100)	
No Yes	790 (72.1) 305 (27.9)	152 (67.9) 72 (32.1)	1.3 (0.94–1.78)
Consumption of raw/undercooked meat	1.093 (100)	224 (100)	
No	423 (38.7)	78 (34.8)	
Yes	670 (61.3)	146 (65.2)	1.2 (0.91–1.67)

**Table 3:** Risk factors for *T. gondii* infection (multivariate logistic regression analysis).

Variable	Multivariate Logistic Regression OR (95% CI)
Age group	
18–29 years	ref.
30–39 years	1.9 (1.13–3.28)
40–49 years	3.2 (1.91–5.35)
50–59 years	4.6 (2.71–7.89)
>60 years	6.8 (3.27–14.24)
Residence area	
Urban	
Suburban	2.2 (1.43–3.34)
Contact with soil	
No	
Yes	1.4 (1.01–1.94)

## Data Availability

All references found eligible in our literature review are included in the article.
